# What does the evidence tell us? Revisiting optimal cord management at the time of birth

**DOI:** 10.1007/s00431-022-04395-x

**Published:** 2022-02-02

**Authors:** Heike Rabe, Judith Mercer, Debra Erickson-Owens

**Affiliations:** 1 Brighton and Sussex Medical School, University of Sussex, Brighton, UK; 2 Neonatal Research Institute at Sharp Mary Birch Hospital for Women and Newborns, San Diego, CA USA; 3 College of Nursing, University of Rhode Island, Kingston, RI USA

**Keywords:** Placental blood, Newborn, Postpartum adaptation, Cord clamping, Cord milking

## Abstract

A newborn who receives a placental transfusion at birth from delayed cord clamping (DCC) obtains about 30% more blood volume than those with immediate cord clamping (ICC). Benefits for term neonates include higher hemoglobin levels, less iron deficiency in infancy, improved myelination out to 12 months, and better motor and social development at 4 years of age especially in boys. For preterm infants, benefits include less intraventricular hemorrhage, fewer gastrointestinal issues, lower transfusion requirements, and less mortality in the neonatal intensive care unit by 30%. Ventilation before clamping the umbilical cord can reduce large swings in cardiovascular function and help to stabilize the neonate. Hypovolemia, often associated with nuchal cord or shoulder dystocia, may lead to an inflammatory cascade and subsequent ischemic injury. A sudden unexpected neonatal asystole at birth may occur from severe hypovolemia. The restoration of blood volume is an important action to protect the hearts and brains of neonates. Currently, protocols for resuscitation call for ICC. However, receiving an adequate blood volume via placental transfusion may be protective for distressed neonates as it prevents hypovolemia and supports optimal perfusion to all organs. Bringing the resuscitation to the mother’s bedside is a novel concept and supports an intact umbilical cord. When one cannot wait, cord milking several times can be done quickly within the resuscitation guidelines. Cord blood gases can be collected with optimal cord management.

* Conclusion*: Adopting a policy for resuscitation with an intact cord in a hospital setting takes a coordinated effort and requires teamwork by obstetrics, pediatrics, midwifery, and nursing.

**What is Known:**

*• Placental transfusion through optimal cord management benefits morbidity and mortality of newborn infants.*

*• The World Health Organisation has recommended placental transfusion in their guidance.*

**What is New:**

*• Improved understanding of transitioning to extrauterine life has been described.*

*• Resuscitation of newborn infants whilst the umbilical cord remains intact could improve the postpartum adaptation.*

## Introduction 

During pregnancy, fetal blood circulates between the fetus and the placenta providing essential nutrients and oxygen. At delivery, infants undergo rapid changes in Circulation and breathing in order to adapt to extra-uterine life. Optimal cord management (OCM), involving waiting several minutes before clamping and cutting the cord has been shown to increase circulatory stability and reduce in-hospital mortality [1–3]. Or if unable to wait, gently milking the cord towards the infant provides some placental transfusion and is preferable over immediate cord clamping (ICC) [3]. The different methods of OCM are listed in Table 1. The World Health Organization (WHO) has recommended delayed cord clamping (DCC) as standard practice at the delivery of all infants but implementation is still variable across health care settings and countries [4, 5]. We provide an overview of current knowledge about OCM and future perspectives.

**Table 1:** Methods of optimal cord management

Method name	Explanation of procedure
Delayed (deferred) cord clamping	Leaving the cord intact for 30–**60** s in preterm infants, at least **3–5 min** in term infants before clamping and cutting the cord
Resuscitation with the intact cord	Leaving the cord intact and starting resuscitation before clamping and cutting the cord
Intact cord milking	Repeated compression and stripping of the cord from the placental side, toward the infant while connected to the placenta after birth
Cut cord milking	Draining the cord by compression and stripping from the cut end toward the infant after clamping and cutting a long segment

## The physiology of placental transfusion

### What cord blood contains

The residual placenta blood returns to the newborn warm (body temperature) and oxygenated. It contains about 15–20 mL/kg of red blood cells which provides the term infant with an adequate iron supply for four to 12 months [6, 7]. There are several million to a billion stem cells providing an autologous transplant which may reduce the infant’s susceptibility to both neonatal and age-related diseases [8]. Progesterone is neuroprotective, and levels in the blood of term infants at birth are higher than the mother’s levels. This likely aids vasodilation and enhances the bodily distribution of the large amount of placental transfusion [9, 10]. In addition, there are numerous additional components such as cytokines, growth factors, and important messengers in cord blood that most likely support and drive the process of transition but are too numerous to discuss here [11].

The full placental transfusion offers high pulmonary artery pressure in the first few hours of life to assist with neonatal adaptation [12]. It is likely that the force of the increased blood volume stimulates multi-organ perfusion for normal organ function, growth, and development. New research reveals that enhanced vascular perfusion causes an organ’s endothelial cells to release angiocrine responses to guide essential functions [13].

#### Circulation

Throughout pregnancy, the fetal blood volume is approximately 110–115 mL/kg of fetal weight [14]. Only ~ 10% of the fetal cardiac output goes to the systemic circulation in the lungs, while 30 to 50% goes to the placenta where gas exchange takes place. In order to change from placental gas exchange to lung air exchange at birth, 45–50% of the newborn cardiac output must rapidly go into the alveolar capillary network in the lungs. Waiting to clamp the cord results in a net transfer of approximately 30% of the fetal-placental blood volume over the first few minutes after birth from the placenta to the neonate. This provides the blood volume needed to fill the alveolar capillary network for the first time and to fully perfuse organs previously supported by the placenta [15].

Classic physiologic studies completed over the past 60 years have documented that placental transfusion results in improved perfusion in the neonate’s respiratory, hematologic, urinary, gastrointestinal, and neurological systems [16–18]. We have proposed that the blood volume received from a placental transfusion increases systemic and regional blood flow and vasodilation in the newborn and aids in normal organ development and health. Immediate cord clamping (ICC) reduces the neonate’s blood volume and may contribute to loss of organ-specific vascular competence [13]. Receiving an adequate blood volume from placental transfusion may be especially protective for the distressed neonate preventing hypovolemia and supporting optimal perfusion to all organs [13, 19].

One of the key features of the normal fetal to neonatal transition at birth is a reduction in the pulmonary vascular resistance (PVR) which allows an increase in pulmonary blood flow and redirects the right ventricular output through the lung instead of through the ductus arteriosus [20]. Credit has been given to lung aeration for decreasing the PVR at birth although the mechanisms causing this has been debated for decades [21]. A new study demonstrated that vagotomy inhibits the previously observed increase in pulmonary blood flow with partial lung aeration [21]. Compared to control newborn rabbits, Lang et al. found that animals after vagotomy had little or no increase in pulmonary blood flow when ventilated with air or nitrogen gas. Using 100% oxygen for ventilation only partially mitigated this effect. This information suggests that the initial dramatic fall in PVR likely does not occur with ventilation and breathing alone and that the vagus nerve, likely stimulated by the increased blood volume, plays a significant role. It appears that with the important task of lowering the PVR, there are multiple overlapping mechanisms to ensure that the transition happens [21].

### Factors that affect the amount and speed of the placental transfusion

**Fig. 1:**
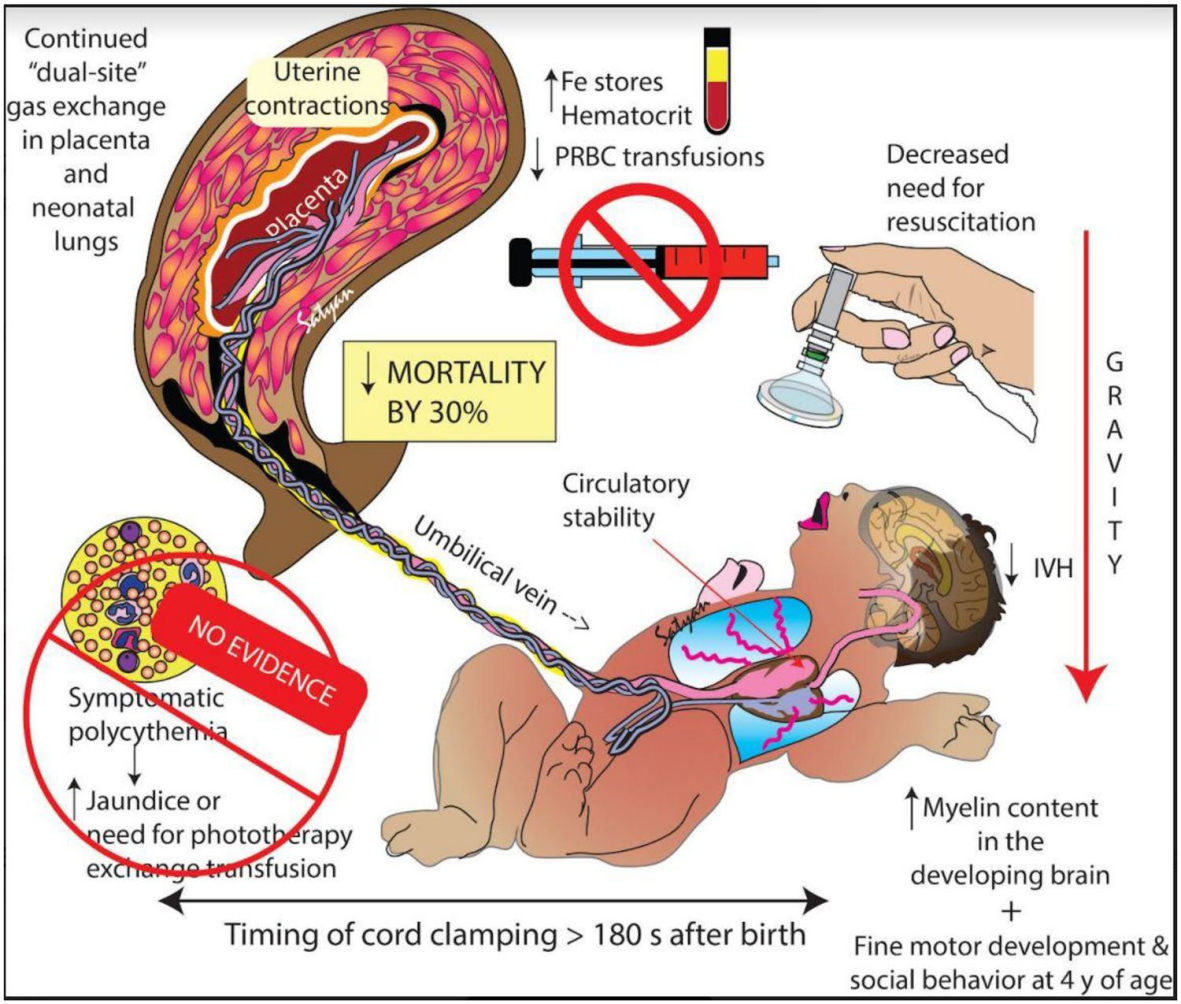
Factors influencing placental transfusion with DCC. Timing of cord clamping, uterine contractions, spontaneous respirations, and gravity influence the magnitude of transfusion. Reported long-term benefits are shown. (Copyright Satyan Lakshminrusimha — used with permission)

**Fig. 2:**
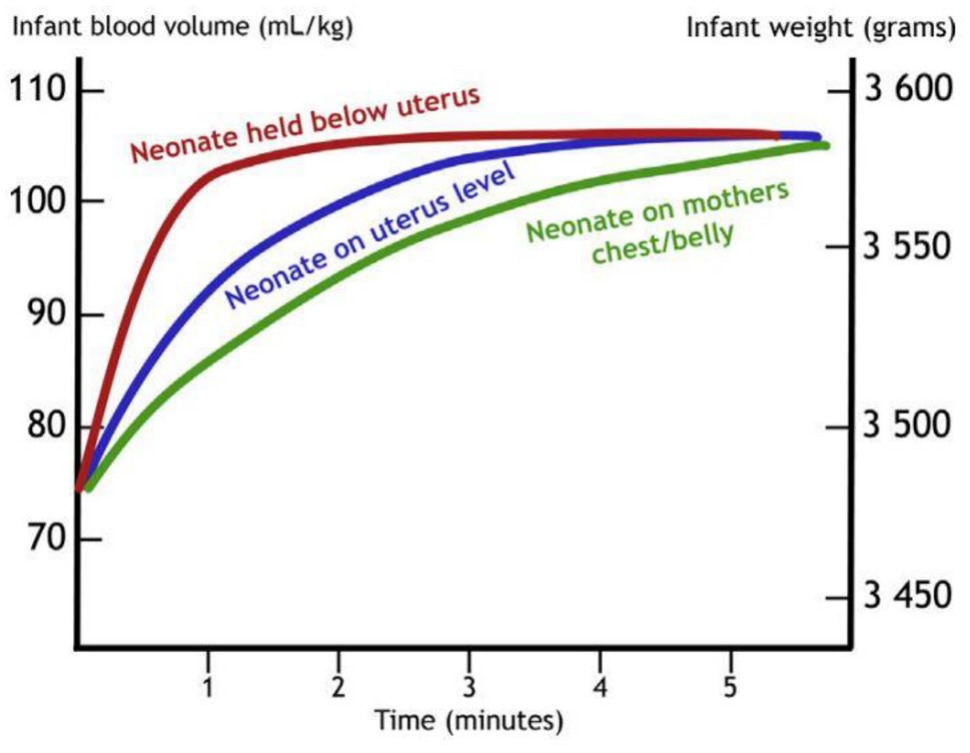
The speed and volume of placental transfusion in relation to time and relative position of the neonate in relation to the placenta (Courtesy of Ola Andersson — used with permission.)

The volume of the transfusion in the term infant receiving DCC is approximately 85 to 100 g or ~ 30 mL/kg [22–24]. For preterm infants, Aladangady reported increased blood volume with DCC at all births, but a smaller increase when born by cesarean section [25]. Factors that affect the amount and speed of the placental transfusion include the timing of umbilical cord clamping, gravity, cord pulsations, uterine contractions, and milking the umbilical cord (UCM) which are discussed below, (see Figs. 1 and 2).

#### Timing of umbilical cord clamping

It is well known that DCC or a delay before clamping increases the amount of placental transfusion to the infant but the optimal time is still controversial. As one delays clamping longer, the neonatal hematocrit at 24–48 h increases [23, 26, 27]. Term infants with a 5-min delay showed an early hematologic advantage compared to infants receiving ICC, without any increase in hyperbilirubinemia requiring phototherapy or symptomatic polycythemia out to 48 h of age [28]. New evidence from a recent landmark study in asphyxic and asystolic lambs found that leaving the cord intact over a 10-min period mitigated the rebound hypertension (by 20–30 mmHg) commonly seen after an asphyxial event [29]. If cord clamping was immediate or only delayed 1 min, the hypertension tended to be worse. This study suggests that the post-asphyxia rebound response may contribute to the brain injury and seizures that often follow severe asphyxia. Prevention of the overshoot may alleviate its contribution to brain injury. Clinical studies (see Table 2) underway to address the question of longer delays in cord clamping with normal and distressed neonates are discussed later under “Ongoing research and future considerations.”

**Table 2:** Current or proposed studies on intact cord resuscitation

Study acronym and country	Proposed *N*	GA (weeks)	Intervention	Cord clamping time, control	Cord clamping time, intervention	Primary outcome	Expected end date
VentFirst (USA) NCT02742454 [75]	940	23–28	CPAP 30–120 s	30–60 s	120 s	IVH, HR, SpO2, Apgar scores ≤ 10 min	2024
PCI-Trial (Italy) NCT02671305 [76]	202	23–29	Resuscitation as needed	Intact UCM × 4	3 min	Composite outcome of severe IVH, CLD or death	2022
ABC2 (Netherlands Trial Registry) NTR7194 [77]	660	24–30	Resuscitation if needed	ICC	Cord clamping when stable*	Intact survival — without IVH or NEC	2024
MINVI – Milking in Non-Vigorous Infants (USA) NCT03631940	1200	35–42	Milking the cord × 4 times	ICC	UCM × 4 before clamping	Admission to the NICU	2023
Baby DUCC (Australia) 12,618,000,621,213 [78]	120	32–41	Resuscitation if needed	ICC	Until 1 min after CO2 detector changes or 5 min	Heart rate at 60 and 120 s	2023
SAVE: Effects of DCC during resuscitation (Sweden) NCT04070560	600	35–42	Resuscitation for non-breathing infants	60 s to resuscitaire	> 180 s with intact cord near mother	Apgar at 5 min	12/2026
CHIC — congenital diaphragmatic hernia intact cord (France) NCT04429750 [51]	180	> 36	Resuscitation of Infants with Congenital Diaphragmatic Hernia	ICC with transfer to Resus room	Intact cord resuscitation on dedicated trolley near mother	Apgar score at 1 and 5 min	No data

#### Gravity

Holding the newborn above the placenta slows transfusion, whereas lowering the newborn hastens transfusion [7, 30]. However, cord clamping at 1 min with the infant on the maternal abdomen can reduce the estimated placental transfusion by 50% [31]. Weight gain (a proxy for amount of placental transfusion) was only 50% of expected amount after two minutes of DCC in a study that weighed infants to compare the effect of gravity. The researchers lowered half the cohort below the perineum and placed the other half on the maternal abdomen [32]. The influence of gravity is illustrated in Fig. 2.

#### Cord pulsations

Pulsations of the intact cord appear to last longer than previously thought according to two recent studies [33, 34]. Using a Doppler, Boere found that over 80% of the infants had umbilical artery flow for over 4 min after birth and 43% still had flow when the cord was cut at 6 min [33]. The flow was pulsatile similar to the infant’s heartbeat and was mainly unidirectional, from the infant to the placenta. DiTommaso, using palpation to examine duration of cord pulsations at vaginal birth, found that the median duration was 3.5 min. They found that infants with a longer duration of cord pulsations had higher birthweights (3530 vs. 3250, *p* = 0.005) and a longer third stage of labor (12 vs 8 min, *p* < 0.001), without any increased risk of postpartum hemorrhage [34]. Both studies suggest that the placenta continues to support the infant longer than previously thought.

#### Uterine contractions

During the few minutes surrounding birth, uterine contractions squeeze blood from the placenta to the infant. However, as the uterus relaxes in-between contractions, blood can flow through the placenta exchanging nutrients and gases for the fetus/infant [35, 36]. This process continues for longer than originally thought and is a valuable asset especially for the infant who is not breathing [33]. Even after the umbilical arteries close, the strong uterine contractions of third stage force more blood to the infant via the umbilical vein, if the cord is left intact.

#### Umbilical cord milking

Although it is not physiologic, milking the umbilical cord two to four times towards the baby has been studied as an alternative to waiting for at least 60 s before clamping the cord [37, 38]. Meta-analyses of studies using UCM show similar benefits to waiting for 60 s, with increased survival by 27% compared to ICC with no difference in major co-morbidities of prematurity [3, 39, 40]. Based on this evidence, many key perinatal learned societies and stakeholders recommend the use of UCM before clamping the cord but only if DCC is deemed not feasible [39, 40].

### Benefits for term and preterm infants

Benefits for term neonates include higher hemoglobin levels, less iron deficiency in infancy, improved brain myelin volume out to 12 months, and better motor and social development at 4 years of age especially in boys [41–43]. For preterm infants, benefits include less intraventricular hemorrhage, fewer gastrointestinal issues, lower transufusion requirements, and decreased mortality by 30% in the NICU and out to two years of age [1, 44, 45]. Perhaps the benefit most familiar with clinicians is the prevention of iron deficiency and anemia in infancy. Both iron deficiency and anemia have a high prevalence in low- and middle-income countries and are associated with perinatal mortality, delayed child mental and physical development, and reduced visual and auditory function [43, 46]. Anemia in infancy contributes to the global burden of morbidity and mortality during the first year of life. OCM can contribute to preventing anemia in the newborn and increased better iron storage up to 12 months of age in term infants [7]. Providers should consider that OCM is free of charge and an easy preventative measure which can be applied in any health care setting, in any gestational age and is strongly recommended by the WHO [4].

### Potential fetal risk conditions

Fetuses who were small for gestational age at birth also benefit from OCM [47, 48]. DCC improves iron stores in SGA infants ≥ 35 weeks at 3 months of age without increasing the risk of symptomatic polycythemia, need for partial exchange transfusions, or morbidities associated with polycythemia. Thus, the WHO recommends providing OCM for them at birth. The same applies to mothers with human immunodeficiency virus infection and low viral load as transmission from mother to fetus is very low [49]. A study of mother-infant pairs with Rhesus-alloimmunisation demonstrated that infants who received placental transfusion at delivery had reduced need for immediate blood exchange transfusion after birth and were successfully managed with phototherapy and blood transfusions as needed [50].

The myth about an increase in jaundice requiring phototherapy has been refuted by recent meta-analyses [1, 2, 23, 44]. Studies on infants with congenital heart disease or diaphragmatic hernia have demonstrated benefits for postnatal adaptation due to their increased need for red blood cells as oxygen carriers. It makes sense to provide them with more of their own blood through placental transfusion at birth [51, 52]. Studies of multiple births have demonstrated feasibility of providing OCM to twins and triplets [53, 54]. Thus, multiple births should not be routinely excluded. The plan for delivery of fetuses with the conditions mentioned above should be considered on an individual basis with a decision about OCM made by an experienced perinatal team ahead of birth.

### Potential maternal/fetal risk conditions

Maternal contraindications of OCM especially focused on DCC have not been formally studied. There are almost no indications for ICC, nor contraindications to OCM. The need for maternal resuscitation in the face of massive, acute hemorrhage would be a rare, justifiable reason to proceed with ICC but cut UCM may still benefit the newborn. A ruptured vasa praevia, snapped cord, or other trauma to the cord vessels, which will result in hemorrhage from the baby, would also be reasons for ICC. In the case of a complete placental abruption where the placenta is delivered at the same time as the baby, it could be held above the baby, with gentle application of pressure to the placenta, and then clamped before the placenta is lowered. Cord milking could also be considered in this situation. A short cord length might interfere with the management of the mother or baby but can usually be addressed with optimal positioning. It should not be considered as an automatic indication for ICC, nor a contraindication to OCM.

### Stabilization and resuscitation with the cord intact

When an infant is born, the intrapartum provider must quickly decide how to manage the umbilical cord. At an uncomplicated birth, DCC is a well-supported, evidence-based practice which facilitates placental transfusion. When an infant is in distress, providers in hospital settings will often practice ICC and transfer the infant to the warmer for resuscitation, away from the mother’s bedside. This practice may lead to increased morbidity and mortality and a disruption in neurodevelopment [44, 55]. The risks of ICC have been studied indirectly as ICC has been the comparator for many studies on DCC and UCM. A list of potential risks is shown in Table 3. The practice of stabilization and resuscitation with an intact cord is not a new idea yet it is infrequently used in hospital settings. Midwives, in out-of-hospital settings, frequently maintain an intact cord after birth and regard the umbilical cord as a lifeline to assist in transition to neonatal life [7, 56].

**Table 3:** Potential harmful effects of immediate cord clamping compared to delayed cord clamping or milking of the cord

Organ system	Effects of immediate cord clamping
Hematology	**↓** RBC Volume, **↓** Hematocrit, **↓** Hemoglobin **↑** Hypovolemia
Body Iron Stores	**↓** Ferritin (out to 4–8 months) **↓** Total Body Iron (at 6 months)
Cardiovascular	**↓** Adaptation **↓** Blood Pressure **↑** Vascular resistance **↓** RBC flow to brain (18%) **↓** RBC flow to gut (15–20%)
Birth weight	**↓** Lighter by 60–101 g
Skin	**↓** Cutaneous perfusion **↓** Peripheral temperature
Renal function	**↓** Renal blood flow **↓** Urine output **↑** Sodium excretion
Respiratory circulation	**↓** Pulmonary vasodilatation

RBC: red blood cells; **↑** increase; **↓** decrease [1–3, 79]

New evidence suggests a distressed infant should be stabilized and resuscitated with an intact cord. This fosters placental transfusion and allows the placenta to continue its important respiratory role in gas exchange and supports volume repletion. A tight nuchal cord and/or a shoulder dystocia are often associated with slow-to-start infants secondary to hypovolemia. The Somersault Maneuver is recommended to release the tight nuchal cord allowing the cord to remain intact [57]. For both nuchal cord and shoulder dystocia, the restoration of blood volume and continued oxygen support from the placenta, alongside Neonatal Resuscitation Program (NRP) and International Liaison Committee for Resuscitation (ILCOR) protocols, help to assist the newborn’s transition [19, 55]. and are important actions which can help protect the newborn’s heart and brain. Resuscitation tables (trolleys) and the accompanying resuscitation equipment have been developed for this purpose [55]. These tables can be moved alongside the mother’s bedside, and the resuscitation can be conducted with an intact cord. Andersson and colleagues (2019) demonstrated in distressed infants, ≥ 35 weeks gestation, that resuscitation with an intact cord resulted in better oxygen saturation levels and Apgar scores [58].

### Practices that may interfere with placental transfusion

Two birth practices, cord blood banking and cord gas collection, imply ICC and challenge the support of an intact cord at birth. Umbilical cord blood banking is the collection of residual placental blood, per parental request, for the purposes of stem cell collection which is then stored in a private or public blood bank. Placental transfusion, via DCC, is not compatible with cord blood banking. In the USA, the American College of Obstetricians and Gynecologists do not recommend the practice of private blood banking nor the interference of the routine practice of DCC [59]. Parents should be informed about the entire contents of cord blood and risks associated with ICC before they make a decision about banking their infant’s blood [7]. 

The practice of umbilical cord gas collection is routine in some hospital settings or may be used judiciously to assess acid–base balance in complex clinical situations. Most providers double clamp the cord immediately after birth and save a section of the cord to be used for analysis. In light of the benefits of DCC, researchers have examined the accuracy of cord gas results comparing ICC and DCC. In a recently published systematic review, it was found that a delay of 2 min in clamping made little difference on the accuracy of cord gas results [60]. Also, cord blood gases may be collected from an intact cord [61].

### Ongoing research and future considerations

There has been increasing interest in stabilization of the preterm or term infant, while the cord is still intact. A list of ongoing or planned studies is provided in Table 2, and Katheria has written an excellent review [62]. We propose that the totality of a placental transfusion is what is important to the baby. It is not just the components — red blood cells, stem cells, cytokines, growth factors, plasma, blood volume, progesterone, or force of the blood for transduction — that make a difference. Collectively, all these pieces working together create the whole process of a normal transition for the neonate. This is a clear biological case where the “whole is greater than the sum of its parts.” If the whole is not considered, it does not work as well and can leave the infant deficient in a variety of ways. This idea may be part of the reason that the uptake of DCC/placental transfusion into clinical practice is slow as placental transfusion does not fit well into the current scientific paradigm of reductionism.

### Recommendations for practice

Support of placental transfusion is recommended as the standard of care at the time of birth, across all birth settings, all modes of delivery and for both term and preterm newborns [4, 7]. This recommendation helps to decrease iron deficiency anemia in infancy and reduces a negative impact on the developing brain [41, 63]. Distressed infants who require resuscitation often have their cords cut immediately and are rapidly transferred to a resuscitaire (warmer), away from the mother’s bedside. [64, 65] Leslie and colleagues (2020) found that US midwives often practiced DCC (98%) and waited for cord pulsations to cease. But these same midwives felt they needed to use ICC in situations which required neonatal resuscitation and/or umbilical cord gas collection often because of institutional policies and time pressures [66]. Emerging evidence suggests that distressed infants require a placental transfusion as much if not more than a healthy newborn[7, 19]

In complex clinical situations that require neonatal resuscitation, new available research suggests the resuscitation can be conducted with an intact umbilical cord managed at the mother’s bedside [55, 67]. This can enhance resuscitation efforts and provide a return of the infant’s own warm, oxygenated blood. When resuscitation with an intact cord is not feasible, milking the cord can be done quickly (several times) within the current NRP and ILCOR standards benefitting infants 28-week gestation or greater [Madar, 2021]. Milking can accelerate the transfer of residual placental blood although not as completely as DCC or an intact cord [55, 68]. Establishing a policy for resuscitation with an intact cord within a hospital setting takes a coordinated effort, logistical considerations and requires multidisciplinary support [55, 65, 69].

One large randomized controlled trial, comparing DCC with UCM, found a significant higher rate of intraventricular hemorrhage with UCM, in infants less than 28-week gestation, but recent meta-analyses of comparative studies found no difference for this outcome [2, 70]. The question remains as to how UCM in infants less than 28-weeks compare to infants receiving ICC differ on variables of IVH and neurodevelopmental follow-up [40].

### Implementing a protocol

It can take significant time (sometimes 10 years or more) for evidence-based approaches to become accepted into clinical practice [71]. In fact, many effective interventions fail to be adopted (60–70%) [72]. This presents significant challenges when trying to translate research findings, such as those from OCM, into practice. Unfortunately, failure to adopt beneficial practices in a timely fashion may lead to unnecessary harm to the newborn [71–73]. Mounting evidence, especially a decrease of mortality in preterm infants, supports the benefits of DCC [45]. This has led to a greater willingness for hospital settings to adopt DCC. Yet, DCC appears to be more easily adopted by providers caring for preterm infants when compared to term infants [74]. Implementation of OCM for all infants requires a step wise approach in planning and implementation [5]. This includes a needs assessment to assess organizational and individual willingness to change. Stakeholders and end users (such as obstetricians, midwives, pediatricians and nurses) must be included in order to reduce reluctance to change and to increase confidence and adherence [5]. A multidisciplinary approach supports teamwork and enhances adoption to change. At this point in the process, any concerns and potential contradictions should be addressed in multidisciplinary discussions prior to delivery. A clinical guideline or policy which is simple, easy to follow, and based on current evidence should be developed with input from all team members and should be updated regularly as new evidence emerges. An example of new emerging evidence is the improvement of neonatal resuscitation by maintaining an intact umbilical cord. This is a novel clinical practice in most hospital settings although has been practiced at out of hospital settings back to the time of Aristotle [75]. Once the decision is made to introduce OCM to the hospital setting, a variety of motivational and educational strategies are available. After implementation, an ongoing monitoring plan is needed to watch compliance rates. Reflection, evaluation, and responsiveness (feedback) will enhance sustainability [5].

## Conclusion

Receiving a placental transfusion is beneficial for all term and preterm infants, including those that are distressed. Placental transfusion plays a major role in neonatal transition by preventing hypovolemia and providing better perfusion to all organs. Adopting resuscitation with an intact cord in a hospital setting will take a concerted effort and team works by obstetricians, midwives, pediatricians, and nurses.

## Abbreviations

DCC: Delayed cord clamping
ICC: Immediate cord clamping
ILCOR: International Liaison Committee for Resuscitation
IVH: Intraventricular hemorrhage
NICU: Neonatal intensive care unit
NRP: Neonatal Resuscitation Program
OCM: Optimal cord management
PVR: Pulmonary vascular resistance
UCM: Umbilical cord milking
WHO: World Health Organization
