# Correction to: What does the evidence tell us? Revisiting optimal cord management at the time of birth

**DOI:** 10.1007/s00431-022-04414-x

**Published:** 2022-02-14

**Authors:** Heike Rabe, Judith Mercer, Debra Erickson‑Owens

**Affiliations:** 1 Brighton and Sussex Medical School, University of Sussex, Brighton, UK; 2 Neonatal Research Institute at Sharp Mary Birch Hospital for Women and Newborns, San Diego, CA USA; 3 College of Nursing, University of Rhode Island, Kingston, RI USA

## Correction to: European Journal of Pediatrics https://doi.org/10.1007/s00431-022-04395-x

In Table 3 of this article, the arrows of data under the column “Effects of immediate cord clamping” were missing. The complete Table 3 is shown as follows:

**Table:** 

**Organ system**	**Effects of Immediate Cord Clamping**
Hematology	**↓** RBC Volume, **↓** Hematocrit, **↓** Hemoglobin **↑** Hypovolemia
Body Iron Stores	**↓** Ferritin (out to 4–8 months) **↓** Total Body Iron (at 6 months)
Cardiovascular	**↓** Adaptation **↓** Blood Pressure **↑** Vascular resistance **↓** RBC flow to brain (18%) **↓** RBC flow to gut (15–20%)
Birth weight	**↓** Lighter by 60–101 g
Skin	**↓** Cutaneous perfusion **↓** Peripheral temperature
Renal function	**↓** Renal blood flow **↓** Urine output **↑** Sodium excretion
Respiratory circulation	**↓** Pulmonary vasodilatation

RBC: red blood cells; **↑** increase; **↓** decrease [1–3, 79]

The original article has been corrected.

